# Viewing multiple sequence alignments with the JavaScript Sequence Alignment Viewer (JSAV)

**DOI:** 10.12688/f1000research.5486.1

**Published:** 2014-10-23

**Authors:** Andrew C. R. Martin

**Affiliations:** 1 Institute of Structural and Molecular Biology, Division of Biosciences, University College London, London, WC1E 6BT, UK

**Keywords:** JavaScript, sequence alignment, protein

## Abstract

The JavaScript Sequence Alignment Viewer (JSAV) is designed as a simple-to-use JavaScript component for displaying sequence alignments on web pages. The display of sequences is highly configurable with options to allow alternative coloring schemes, sorting of sequences and ’dotifying’ repeated amino acids. An option is also available to submit selected sequences to another web site, or to other JavaScript code. JSAV is implemented purely in JavaScript making use of the JQuery and JQuery-UI libraries. It does not use any HTML5-specific options to help with browser compatibility. The code is documented using JSDOC and is available from http://www.bioinf.org.uk/software/jsav/.

## Introduction

Viewing multiple sequence alignments (MSAs) is a fundamental requirement in the analysis of protein sequences, allowing us to visualize conservation across protein families as well as unusual features of particular sequences. As a result, there are a plethora of tools for viewing MSAs. These range from tools which provide attractive printed outputs, through standalone graphical tools — either operating-system dependent, or independent — to web-based viewers.

Two of the earliest tools were HOMED ^1^ and MALIGNED ^2^ written for VAX/VMS workstations. Neither seems to be actively maintained or easily available any more. Other early viewers include GeneDoc ^3^, BioEdit, Seaview ^4^ and DCSE ^5^ which is part of the RnaViz package for visualizing RNA secondary structures ^6^, but which can be used for protein sequence alignments. A problem in writing graphical software is the operating-system dependency of many graphics libraries. CINEMA ^7^ was probably the first sequence alignment viewer and editor implemented in Java, a platform independent programming language allowing graphical user interfaces (GUIs) to run on any operating system. It has now been rewritten in C++ and is part of UTOPIA ^8^. Other software includes MPSA ^9^, ANTHEPROT ^10^ and ClustalX ^11^, a GUI for the ClustalW multiple sequence alignment program, providing an integrated environment for aligning sequences and analyzing results. Clustal Omega is the most recent version, but at the time of writing only has a command line interface — a beta version of a GUI is due to be released soon.

More recent developments include the Protein Family Alignment Annotation Tool (PFAAT) ^12^ designed specifically for family analysis and incorporating residue annotation tools as well as integration with Jmol for protein structure display. Like early versions of CINEMA, PFAAT is implemented in Java for operating system independence. CLC Viewer is a recent free package written in Java which contains a number of integrated tools and acts as a core product for adding other features through a commercial version. A more complete list of MSA viewers is available on the web at http://en.wikipedia.org/wiki/List_of_alignment_visualization_software.

Probably the most popular of the available tools is Jalview ^13^ which is available in two versions: a standalone Java application which provides many tools and facilities, and as a ’light’ version (JalviewLight) — a Java applet that can be embedded in a web page. The latter responds to the need for web site developers to be able to embed MSA visualization.

However, in recent years there has been a gradual move away from using Java applets in web development. Java creates an additional layer of software (including additional memory consumption) and modern browsers enforce much more caution in running Java applets to avoid security threats. This can result in user irritation with having to accept various pop-up warnings and/or configure security settings, often having to repeat this process when there is a software update. It is not uncommon for Java applets simply to fail to run, perhaps because users do not understand what settings need to be changed. New HTML features such as the HTML5 Canvas, and powerful JavaScript libraries such as Bootstrap, JQuery and JQuery-UI that provide an easier syntax for accessing elements of a web page together with new widgets such as sliders and drag-and-drop support, have overtaken Java as the method of choice for creating interactive web sites with complex requirements. Such features are used widely by popular web sites such as Google Mail, Google Docs, Twitter and Facebook. Illustrating this trend, the Jmol structure viewer has recently been reimplemented in JavaScript as JSmol ( http://sourceforge.net/projects/jsmol/). Consequently, over the last couple of years, a small number of JavaScript-based sequence and alignment viewers have started to be developed. These include MODalign ^14^, Alignment-Annotator ^15^, SnipViz ^16^, and Sequence ^17^, a component of the BioJS library ^18^. In addition, there is an intention to port JalviewLight to JavaScript. The available programs are briefly reviewed:

**MODalign** is part of the MODexplorer package ^19^, a web site for protein modeling, but does not appear to be available as a download for use in other web sites.

**Alignment-Editor** is part of a more complex system, STRAP. It uses a Java server-side interpreter, Alignment-to-HTML ^20^, which parses the STRAP scripting language and creates an alignment in a form that can be rendered in Web browsers. The server-side element allows tasks such as sequence retrieval, computation of alignments and communication with BioDAS-servers. The rendering system includes a selection of coloring schemes, highlighting of conserved and variable positions in the alignment, reordering and deletion of sequence by drag-and-drop, and residue annotation as well as links to 3D visualization and sequence groups. It exploits JavaScript and HTML5 using the HTML5 canvas to draw helices and other visual elements. However the JavaScript visualizer does not appear to be available by itself. The description of Alignment-Editor ^15^ suggests that alignments should be prepared using the full Java system and the final alignment can then be downloaded as HTML files in a ZIP archive. The software is licensed under the GPL and available from the authors on request, or a desktop version of STRAP can be downloaded or run using Java WebStart. While in principle possible, no simple documentation is provided to enable the client-side JavaScript/HTML5 viewer to be used without the server side software.

**SnipViz** is a compact and lightweight component designed for display of multiple versions of gene and protein sequences — i.e. essentially identical sequences with mutations. It provides a very simple clean display focused around both DNA and protein sequences allowing very long sequences through a scrolling mechanism which also shows a small box on a representation of the complete sequence to show the relative position within the complete alignment. It also allows display of phylogenetic trees stored in Newick format. Note that SnipViz should not be confused with SNPViz ^21^.

**Sequence** is a BioJS component for visualizing sequences rather than alignments. It only provides very simple views with no choices of coloring schemes, although it does provide very flexible highlighting of regions within a sequence.

JSAV (JavaScript Sequence Alignment Viewer) is a novel JavaScript component that adds to this list. The primary motivation for implementing a new tool was for development of our abYsis antibody database ( http://www.abysis.org/), where we required a simple JavaScript component that would enable us to display a set of aligned sequences, sort and select sequences in that alignment for further analysis, and highlight regions of the alignment corresponding to the CDR loop regions of antibodies. Consequently the requirements were as follows: (i) a very simple-to-use lightweight component that can easily be dropped into a web site; (ii) provision of flexible coloring schemes and ’dotifying’ alignments (replacing repeated residues with dots); (iii) the ability to sort sequences — based both on complete sequences or regions of sequences (such as a CDR loop or framework region of an antibody); (iii) the ability to remove sequences from the alignment; (iv) the ability to highlight regions in the alignment and to display a consensus sequence; (v) the ability to export a selected set of sequences in FASTA format; (vi) the ability to submit a selected set of sequences to another web site or to client-side JavaScript code for further processing. Table 1 lists the availability of these and other features in different tools.

**Table 1.** Summary of availability of features in various JavaScript sequence alignment display tools as described in their respective papers and web sites. Note that the MODAlign web site was unavailable at the time of writing so capabilities have been judged purely on what is published in the paper. † Not available for use in other web sites. ‡ Should be possible, but not designed to be used in this way. * Submission to Modeller only. § Automatically highlights mutated residues. ¶ Highlights columns of residues conserved at a specified level rather than displaying a consensus.

	JSAV	MOD- align	Alignment- Editor	SnipViz	Sequence
Simple, lightweight component	✓	†	‡	✓	✓
Flexible coloring schemes	✓	✓	✓		
Dotifying	✓				
Automatic sequence sorting	✓				
Manual sequence sorting			✓		
Remove sequences	✓		✓		
Highlight regions	✓		✓	§	✓
FASTA export	✓	✓	✓		✓
Submission to another site	✓	*			
Submission to JavaScript	✓				
Display consensus sequence	✓	✓	¶		
Display secondary structure		✓	✓		
Alignment editing		✓			
Link to structure viewer		✓	✓		
Display phylogenetic tree				✓	
Optimized for very long sequences				✓	

## Software tool

JSAV allows the end-user to modify the display in a number of ways. The web site provider has control over which of these is available to the end user. First, the sequences can be sorted — the code selects the most representative sequence, displaying that at the top of the alignment followed by the most similar sequence and so on. By default, sorting is performed across the whole sequence, but a two-handled slider allows the range of positions on which the sort is based to be modified. Different coloring schemes are available duplicating those provided in Jalview. The alignment can also be ’dotified’, replacing residues repeated between sequences with dots in order to emphasize amino acid differences. Coloring of dotified residues can also be switched off or on. Sequences can be selected and deleted from the alignment; a consensus sequence can be displayed at the bottom of the alignment and updates automatically when sequences are deleted. The complete set of sequences, or a selected subset, can be submitted to another web site, or passed to another JavaScript function for integration with other tools. Tooltips are provided for each option and all options are documented in detail on the web site.

JSAV is implemented purely in JavaScript. Code is managed using GitHub ( http://www.github.com) and documented using JSDOC ( http://usejsdoc.org). JSAV employs the JQuery library to ease access to elements of the HTML that it generates and uses JQuery-UI to implement a two-value slider that is used to specify a range of positions in the alignment. As input, the code requires an array of JavaScript objects which contain two elements: a unique identifier for a sequence and the sequence itself — all sequences must be pre-aligned. Secondly a set of options can be provided. Options fall into two classes: those that control the (initial) display and those that control facilities available to the end user of a web site to modify the view of the MSA. Options that control the display include: (i) ranges of alignment column positions to be highlighted; (ii) the color scheme to be used; (iii) whether the sequence should be dotified and whether repeated residues should be colored; (iv) whether a consensus sequence should be displayed; (v) whether a FASTA export button should be available and the label for that button; (vi) the URL and label for a button to allow selected sequences to be submitted to another web site; (vii) a JavaScript function name and label for a button to allow selected sequences to be processed by code written by the web site developer; (viii) whether plain tool tips should be used rather than those provided by JQuery — more attractive tooltips available with the ’tooltipster’ package are also supported. Options that control how the end-user can manipulate the display include: (i) whether the alignment should be sortable and, if so, the width and height of the slider used to select a region for sorting; (ii) whether selection check-boxes should be displayed next to each sequence; (iii) whether sequences can be deleted from the alignment; (iv) whether the user should be able to toggle the dotifying of the alignment; (v) whether the user should be able to toggle not coloring dotified residues; (vi) whether a pull-down should be displayed to select color schemes.

**Figure 2.**
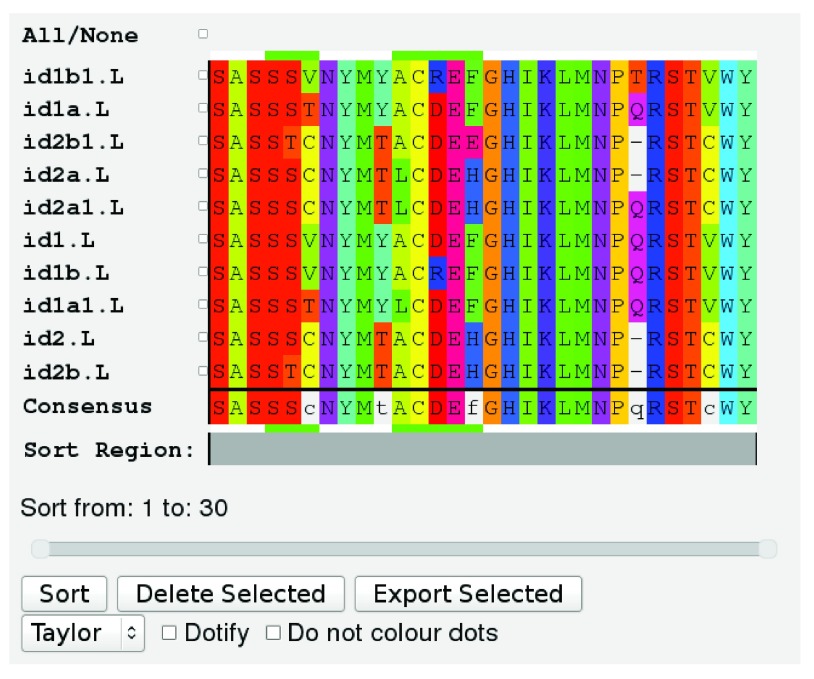
A typical JSAV alignment view.

The sequence alignment is rendered as a table and all display of colors and layout is achieved through Cascading Style Sheets (CSS). Consequently, a web-site developer can easily add a new color scheme by modifying the CSS file and setting an option to specify available color scheme names. The number and size of sequences in the MSA is limited only by the memory available to the web browser. A brief extract of sample code is shown in Figure 1 with the results shown in Figure 2.

**Figure 1.** Example code illustrating the creation of a JavaScript array of sequence objects, the options and the call necessary to create the alignment viewer. var MySeqs = Array();
MySeqs.push({ id :"id1b1.L", sequence :"SASSSVNYMYACREFGHIKLMNPTRSTVWY"}); 
MySeqs.push({ id :"id1a.L",  sequence :"SASSSTNYMYACDEFGHIKLMNPQRSTVWY"});
MySeqs.push({ id :"id2b1.L", sequence :"SASSTCNYMTACDEEGHIKLMNP-RSTCWY"}); 

var MyOptions = Array();
MyOptions.sortable = true;
MyOptions.selectable = true; 
MyOptions.deletable = true; 
MyOptions.toggleDotify = true;
MyOptions.toggleNocolour = true;
MyOptions.consensus = true;
MyOptions.selectColour = true;
			 
printJSAV(’sequenceDisplay’, MySeqs, MyOptions);

JSAV deliberately avoids making use of HTML5 to maximize browser compatibility. It is known to work with modern browsers including Firefox V32.0, Chrome V37, Konqueror V4.13.3, Safari V5.1.7 and Explorer V10.0 on Linux, Mac and Windows platforms and is known to work on versions of Firefox as old as V9.0.1. JSAV has been developed and tested using JQuery V1.10.2 and JQuery-UI V1.10.4, but it only uses the JQuery HTML element selection mechanism and tool tips and the two-handled slider component from JQuery-UI and consequently would be expected to work with much earlier versions.

## Conclusions

Viewing multiple sequence alignments is a fundamental requirement of protein sequence analysis. With that large amounts of Bioinformatics work being performed over the web, there is a clear need to be able to embed MSA viewers within web pages. The recent move away from Java in favor of JavaScript has driven a need for MSA viewing tools written in JavaScript. While four other tools have been made available, two do not appear to be available as simple components that can be used by a web developer to provide sequence alignments (MODalign and Alignment-Editor). Of the other two, SnipViz has only very limited facilities and is designed for viewing SNPs in alignments of very similar sequences while Sequence is only designed for displaying single sequences and not MSAs.

Consequently JSAV fills this gap, providing a very simple-to-use component that can just be passed an array or pre-aligned sequences, but which also has the flexibility to allow manipulation of the way in which the MSA is displayed. JSAV provides a number of features that appear to be absent from any of the other tools including dotifying alignments, automatic sorting of sequences (including limiting the sort to a region within the MSA), and submission of selected sequences to other web sites or to other JavaScript code.

Future directions are likely to include modifying the JSAV component to become part of BioJS ^18^ and linking JSAV with JSMol for structure visualization.

## Software availability

### Software access

The software may be downloaded from http://www.bioinf.org.uk/software/jsav/ where demonstrations, including the ability to upload your own MSA, are available together with full documentation implemented with JSDOC.

### Latest source code

http://www.github.com/AndrewCRMartin/JSAV

### Archived source code as at the time of publication

http://www.dx.doi.org/10.5281/zenodo.11980 ^22^

### License

GNU GPL License. Commercial licences available on request.
