# Supplementary material for: Viewing multiple sequence alignments with the JavaScript Sequence Alignment Viewer (JSAV)
Source: F1000Res. 2014 Oct 23;3:249. [Version 1] doi: 10.12688/f1000research.5486.1 (PMC4304231; doi:10.12688/f1000research.5486.1)
Supplement: Peer review history

# Peer review: Referee response for version 1

**DOI:** 10.5256/f1000research.5856.r6667

**Published:** 2014-12-03

**Authors:** Robert Davey

**Affiliations:** 1 Genome Analysis Center, Norwich Research Park, Norwich, UK

I, Robert Davey, promise:

i) to not hide behind a screen of anonymity

ii) to be open and honest with you (the authors) at all times

iii) to be constructive in my criticism

iv) within the rules given to me by the journal, to assist you in every way I ethically can to get your manuscript published, by providing criticism and praise that is valid and relevant

---

The manuscript describes JSAV, a web-browser based multiple sequence alignment viewer that provides similar but expanded features to previous work in this domain.

The tool's API and documentation is relatively clear and simple, and the source code is freely available. It also uses standard libraries such as jQuery to provide a consistent experience across browsers, which is a good sign for compatibility and user experience.

The authors have given a coherent summary of previous tools, including quite old and standalone solutions for completeness. The authors even go as far as to note potential confusion in similarly named tools ("Note that SnipViz should not be confused with SNPViz.") which is a welcome attention to detail.

The compatibility argument is sound, but did the authors attempt to develop an HTML5 version of JSAV? What would be the benefits over pure Javascript? In a similar vein, JSAV seems to be responsive and functional with a relatively small number of sequences, and therefore could well be a very useful component for online MSA browsing, but the authors do not mention cases whereby far more complex MSA processing may hit the limits of what JSAV can provide. Tools such as JalView are able to harness "heavy lifting" aspects of the Java language. Where do JSAV's abilities end and a desktop tool's features begin? How might JSAV approach these issues?

Specific comments:

- Some sentences are a little long and unwieldy, and could benefit from some reworking, e.g. "New HTML features such as the HTML5 Canvas, and powerful JavaScript libraries such as Bootstrap, JQuery and JQuery-UI that provide an easier syntax for accessing elements of a web page together with new widgets such as sliders and drag-and-drop support, have overtaken Java as the method of choice for creating interactive web sites with complex requirements."
- The sentence "have started to be developed" is clumsy. Consider "are being developed", or "have been developed". The tools in question have been released.
- The list of display options ("Options that control the display include") produces an overly long paragraph of text. Consider changing this to an indented numbered/bullet list or a table for ease of consumption.
- "Bioinformatics" mid-sentence should not be capitalised.
- "The recent move away from Java in favor of JavaScript" - this sentence should be qualified with a context. Java is very much still used for web application backends, but as the authors previously state, Javascript has become the most popular language recently for client-side rendering and processing.

I have read this submission. I believe that I have an appropriate level of expertise to confirm that it is of an acceptable scientific standard.

# Peer review: Referee response for version 1

**DOI:** 10.5256/f1000research.5856.r6666

**Published:** 2014-11-12

**Authors:** David Martin

**Affiliations:** 1 Biological Chemistry and Drug Discovery, College of Life Sciences, University of Dundee, Dundee, UK

Software technical notes are useful contributions to the literature and the state of the art but difficult to review. One ends up assessing the functionality of the software and how readily it can be used for the purpose intended. Does it afford a useful extension to the state of the art, or can it mislead? Can it be readily implemented in workflows or websites (in this case).

In general this is a very welcome contribution. The software is easy to incorporate and provides a useful way to visualise sequence alignments. Having said that, there are minor quibbles, the most serious of which is the display of dotted residues. This is of interest where there are a few specific changes and makes them very easy to spot. However, the implementation in this software appears to be somewhat counter-intuitive. The usual method is for differences to a consensus to be shown, with sequences that are identical represented by the dots. In the implementation in the paper, the dots refer to it being the same as the residue above, where all the meaning is lost if colour is not shown. That is somewhat of a *caveat emptor*, but care should be taken to ensure that such a representation matches the convention expected by the user.

It would be useful also to include some form of export for a visualisation, either through a direct export or through integration with a more powerful package such as Jalview.

In summary it is a useful tool for representation of short alignments and as such will no doubt find utility, I can certainly see uses for it.

I have read this submission. I believe that I have an appropriate level of expertise to confirm that it is of an acceptable scientific standard.

# Peer review: Referee response for version 1

**DOI:** 10.5256/f1000research.5856.r6487

**Published:** 2014-10-23

**Authors:** Christoph Gille

**Affiliations:** 1 Department of Biochemistry, Charité – University Medicine Berlin, Berlin, Germany

I tried JSAV in a few browsers including Opera, Chrome and Konqueror and in all browsers the alignment was well displayed.

The tool is fast even when alignments with 40 sequences and more are submitted.

It is a clean, robust and simple solution for displaying alignments in web pages.

With JSAV, it is very easy to include rendered alignments in web pages.

Innovative is the sequence sorter which allows sorting sequences by local sequence similarity.

The row header runs out of the screen if the alignment is scrolled horizontally. Also there is no wrapping of long sequences as in Jalview. This matters only in case of sequences comprising more than about 100 residues.

Sequence and residue annotations known from Jalview, Pfaat and CLC seem not to be implemented in JSAV.

There are some minor issues concerning the GUI:

1.  The leading greater-than signs from the fasta header is written in front of the sequence name. 
2. The check-box Do-not-color-dots could be deactivated if the check-box Dotify is not activated.
3. The sequences are selected/deselected for deletion or export using check-boxes. Multiple selection of a range of sequences via Shift-left-click does not work. For alignments comprising only a few sequences, however, this does not matter.

The text is well written and the advantage of JSAV over other tools becomes clear: It is a simple Java script library for basic alignment visualization which does not require any other server program.

The table 1 compares existing tools and is very informative. Interesting is the last table row telling that SnipViz allows long sequences. This could be discussed more in the text since this exemplifies a general performance problem of JS compared to C++ or Java: I assume that in all cited JS based tools with the exception of SnipViz, document-object-model-elements (DOM elements) are created for all residues. For large alignments, ten thousands of elements need to be created which takes long time and uses up much memory. With Java, however, only the visible part of the alignment needs to be drawn (Cinema, Pfaat, Strap) which is much faster and has a minimal footprint. I assume that SnipViz only renders the visible portion of the alignment?  This would explain why the alignment cannot be scrolled softly. This kind of discussion would help the reader to understand that using JS for alignment visualization is still technical challenge and one cannot expect the full functionality of CLC workbench or Jalview in a JavaScript library like JSAV.

Alignment-Editor should read "Alignment-Annotator". This tool should have a mark in the table rows Phylogenetic-tree and Submission-to-another-site.

The authors emphasized the program feature of annotations in various cited tools and could provide a row in the table for this important feature.

It would be great if annotations could be implemented in future releases of JSAV.

I have read this submission. I believe that I have an appropriate level of expertise to confirm that it is of an acceptable scientific standard, however I have significant reservations, as outlined above.
